# Long-term outcomes of LT4/LT3 combination treatment for persistent hypothyroid symptoms

**DOI:** 10.1530/ETJ-24-0275

**Published:** 2025-03-04

**Authors:** Birte Nygaard, Christian Zinck Jensen, Mads Jorsal, Bjarke Røssnes Medici, Rudi Steffensen, Allan Carle, Jeppe Lerche la Cour

**Affiliations:** ^1^ Herlev Hospital, Department of Endocrinology, Herlev, Denmark; ^2^ University of Copenhagen Faculty of Health and Medical Sciences, Department of Clinical Medicine, Copenhagen, Denmark; ^3^ Division of Translational Endocrinology, Department of Endocrinology, and Internal Medicine, Herlev, Denmark; ^4^ Aalborg Universitetshospital, Department of Immunology, Aalborg University Hospital, Aalborg, Nordjylland, Denmark; ^5^ Aalborg University Hospital, Department of Endocrinology, Aalborg, Denmark; ^6^ Hvidovre Hospital, Department of Endocrinology, Copenhagen University, Hvidovre, Denmark

**Keywords:** hypothyroidism, liothyronine, quality of life

## Abstract

**Objective:**

Patients are increasingly using and requesting LT4/LT3 combination treatment for persistent hypothyroid symptoms, but the efficacy and side effects of long-term therapy remain largely unexplored. This study aimed to describe the patient group experiencing a long-lasting impact of LT4/LT3 and evaluate their quality of life (QoL) and hypothyroid symptoms.

**Method:**

We performed a cross-sectional study of 66 hypothyroid patients who had previously initiated LT4/LT3 combination therapy. The patients were grouped by current treatment into patients still receiving LT4/LT3 treatment (T3 responders) and patients who had discontinued LT3 treatment due to lack of effect (T3 non-responders). ThyPRO was used to evaluate QoL, and a validated symptom score was used to assess hypothyroid symptoms. The paper describes a real-life study that depicts unsatisfied patients as they are met in an outpatient clinic.

**Results:**

The participants had a median age of 56 and had initiated LT4/LT3 combination therapy 5.4 years ago. Fifty-four patients still received LT4/LT3 therapy and 12 patients had discontinued LT3 treatment due to lack of effect. Patients in the T3 responder group experienced a QoL comparable to the background population. Surprisingly, symptom scores in the T3 responder group were at the same levels as seen in Danish females with overt hypothyroidism. Thyoid stimulating hormone (TSH) in the T3 responder group was less than 0.4 mU/L in 38% of patients, indicating overtreatment.

**Conclusion:**

LT4/LT3 treatment was well-tolerated with no side effects and high QoL, but patients still experienced many symptoms.

## Background

Hypothyroidism is a common disease, and it is well-known that patients treated with levothyroxine (LT4) often experience persistent hypothyroid symptoms despite being considered ‘well-treated’ according to thyroid function blood tests (1, 2). Population-based studies have shown that 34% of LT4-treated hypothyroid patients have a decreased quality of life (QoL) compared to 26% of the general population. In addition, depression is seen in 18% of LT4-treated patients compared to 12% in thyroid healthy individuals (3). Furthermore, LT4-treated patients have a higher risk of receiving disability pensions and having lower incomes (4).

The thyroid gland primarily produces thyroxine (T4) with a half-life of 7 days. Through the action of a deiodinase enzyme, T4 is converted to triiodothyronine (T3), which has a shorter half-life of 1 day and is more potent. One hypothesis is that some patients solely treated with LT4 may lack the T3 produced directly in the thyroid gland, as the thyroid and hypothalamus cells have a more efficient conversion of T4 to T3 compared to other tissues. This suggests that an LT4-treated patient may still experience ‘tissue’ hypothyroidism despite having normal TSH levels on hypothalamic and pituitary evaluation (2).

The use of liothyronine (LT3) in the treatment of specific groups of hypothyroid patients with persistent symptoms as an additional choice alongside LT4 is controversial. In 2009, our group published a blinded cross-over study evaluating LT4/LT3 combination therapy in a selected group of hypothyroid patients (all having a s-TSH >20 at the time of diagnosis) (*n* = 59) with persistent symptoms. It showed that patients on LT4/LT3 combination therapy reported better QoL scores in 7 out of 11 scales using the SF-36 QoL score. In that study, 49% of the patients preferred combination therapy, 15% preferred LT4 monotherapy and 36% had no preference (*P* = 0.002) (5). Similarly, in an analysis of treatment preference, including data from five cross-over trials (*n* = 228), 48% preferred LT4/LT3 combination treatment, 27% preferred LT4 monotherapy and 25% had no preference (1).

A meta-analysis published in 2022, which included 18 studies, concluded that there is still no evidence of the effects of LT4/LT3 combination therapy judged by QoL and depression scores using SF-36, GHQ, BDI, POMS or SCL-90 questionnaire items (6). In addition, two recent high-quality randomized-controlled trials showed no difference between LT4 and LT4/LT3 combination treatment (7, 8). However, a significant number of patients continue to report persistent symptoms, leading clinicians to use LT4/LT3 combination therapy. In an online survey by the British Thyroid Foundation (a patient society), 78% of respondents described poor QoL (9).

There are a few register-based studies on the long-term effects and side effects of LT4/LT3 combination therapy. Still, they all have limitations, such as not establishing correlations between T3 dose or TSH levels and side effects (10, 11, 12, 13). Furthermore, a more detailed description of the patient’s experience of the impact of LT3 treatment and data evaluating the persistence of the treatment effects on QoL and hypothyroid symptoms over time is still lacking.

Patients request personalized treatment, and thyroid experts strive to find safe ways to meet these requests. However, tools to identify which patients should be treated and to find the optimal treatment approach are still missing.

This study aimed to describe the patient group receiving LT4/LT3 combination therapy in a real-life study that depicts unsatisfied patients as they are met in an outpatient clinic to evaluate the long-lasting influence of LT4/LT3 treatment on QoL and hypothyroid symptoms, estimate side effects (such as atrial fibrillation, osteoporosis and general symptoms) and assess changes in medication.

## Methods

### Design

The study was a cross-sectional study of patients who had previously initiated LT4/LT3 therapy. This study describes the secondary outcomes of the study Danish Ethical Committee: journal-no H-1704532.

The primary outcome was to test whether specific genetic variations could predict which patients would benefit from LT4/LT3 treatment. We were not able to include enough patients to answer this question. The results related to genetic variants are provided in Supplement I (see section on Supplementary materials given at the end of the article).

### Patients

Patients were recruited from the Department of Endocrinology, Herlev Hospital, University of Copenhagen. They had initiated LT4/LT3 treatment, intending to follow the ETA guidelines on LT4/LT3 combination therapy (1). Written consent was obtained from each patient after fully explaining the purpose and nature of all procedures used.

Patients were recruited from the following two populations.

**Population 1**. Hypothyroid patients with persistent symptoms who initiated LT4/LT3 therapy in the period 2012–2014 were consecutively recruited from the outpatient clinic at the Department of Endocrinology, Herlev Hospital. Data were published in 2017 and 2018 (14, 15). 113 patients were invited (46 included).

**Population 2**. Screening of patients treated with LT4/LT3 for at least 1 year or who have tried LT4/LT3 therapy with no effect in the outpatient clinic during 2021 – 34 patients invited (20 included).

Hypothyroidism was defined as sustained elevated serum TSH before treatment. Patients were invited to start combination therapy if they complained of persistent symptoms (tiredness, decreased memory and cognitive function, muscle and joint symptoms and weight gain).

### T3 responders versus T3 non-responders

The patients were grouped by current treatment into patients still receiving LT4/LT3 treatment as T3 responders and patients who had discontinued LT3 treatment due to lack of effect as T3 non-responders.

### Patient history and previous blood tests

At the visit, the patients answered a questionnaire regarding comorbidity (stress, depression, osteoporosis and cardiac disease including hypertension, arthritis and cancer), medication and possible side effects of LT3 (heart, gastrointestinal, weight, sleep and psychiatric disease). Previous thyroid function tests (serum TSH and T4) and thyroid hormone substitution doses were verified from a digital journal system.

### QoL scores and symptoms score

At the visit, patients filled out QoL and hypothyroid symptoms scored questionnaires.

QoL was measured using ThyPRO (16, 17). We used the nine subscales from ThyPRO. Each score was constructed by summating relevant items and linear transformation to a range of 0–100, where 100 indicated the most symptoms/impact on QoL (17). QoL scores were compared to historical data from the general population in patients at a median age of 50 years (Q1–Q3: 37–62-years-old) (18) to make Fig. 2.

### Symptom score

We also calculated alternative symptoms score, where the presence of a symptom was coded as a binary variable (yes/no) (some questions were similar to the ones included in the ThyPRO questionnaire). This score consists of a previously validated score list of 13 hypothyroidism-associated symptoms (tiredness, dry skin, mood lability, constipation, palpitations, restlessness, wheezing, globulus sensation, difficulty swallowing, hair loss, dizziness and anterior neck pain) (19, 20, 21). Results are presented as % of patients who have the symptom. Symptoms scores were matched by age and gender of patients having overt hypothyroidism (*n* = 108) and euthyroid controls (*n* = 216) included in the Dan Thyr study (19, 20, 21) to make Fig. 3.

### TSH measurement

At the visit, blood was drawn and plasma TSH (reference level: 0.4–4.8 mU/L) was determined in the local laboratory by a two-site chemiluminescent immunometric assay using a Siemens Atellica®IM Analyser. The coefficient of variation was 2.5 and 5% at TSH levels of 1.04 and 10.7 mU/L, respectively. An electrocardiogram was measured as standard procedure.

### Statistical analysis

Data are presented as *n* (%) or medians and ranges. Groups were compared using the chi-squared test or ANOVA. Bonferroni corrections were applied when relevant.

### Ethics

The study was approved by the Danish Ethical Committee: journal-no H-1704532.

## Results

### Study population

**Figure 1:**
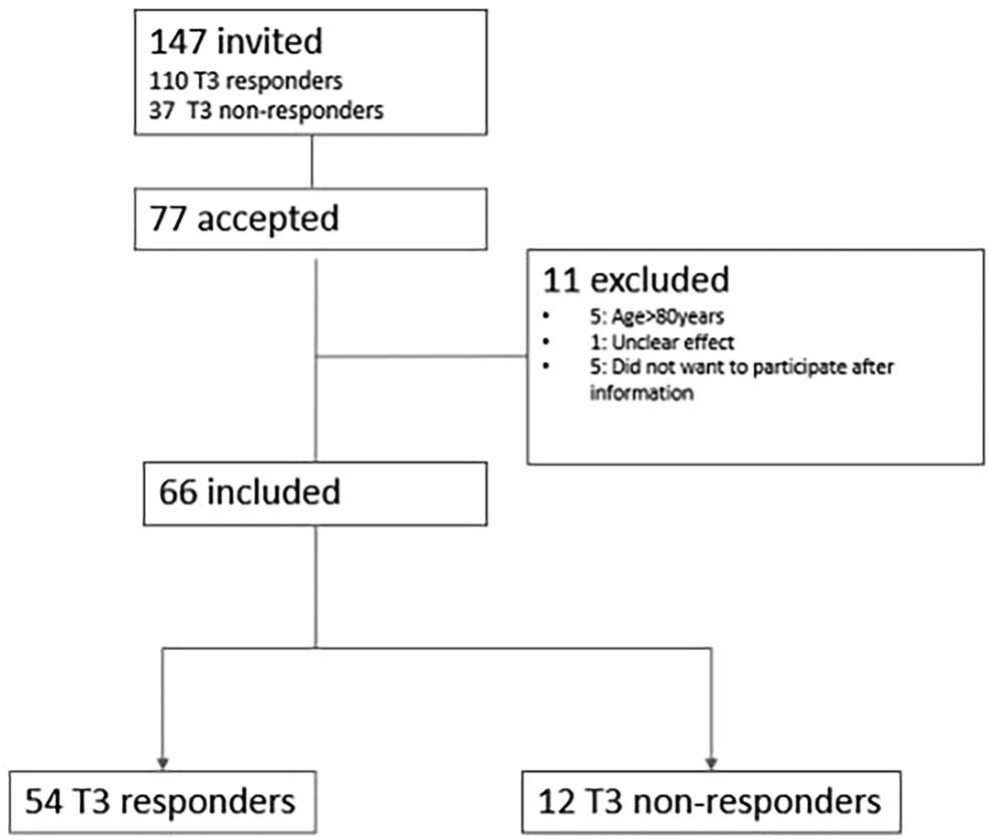
Flow diagram of 147 patients previously initiating LT4/LT3 therapy due to persistent hypothyroid symptoms on LT4 monotherapy.

147 patients identified from two designated cohorts from the Herlev University Hospital were invited. Of these, 110 were registered as T3 responders (evaluated at least 12 months after initiating LT4/LT3 treatment) and 37 were registered as T3 non-responders when the patients were dismissed from the outpatient clinic. Of these, 66 patients (64 female) initiating LT4/LT3 therapy between 2010 and 2019 were included (see Fig. 1).

The median age of the patients was 56 years, ranging from 28 to 77 years. Before the study, they had initiated LT4/LT3 therapy for a median of 5.6 years (range: 0.7–11). Comorbidities (during the past ten years) were seen in a total of 49 (74%) patients: 17% had experienced depression, 46% had had a period of stress (34% needing sick leave), 3% had known osteoporosis and a total of 16% had had cancer (breast, bladder, skin, uterus, renal, lung, colon and melanoma) (Table 1). Of the included patients, 41 were initially diagnosed as spontaneous autoimmune hypothyroidism; five had postpartum thyroiditis; two had subacute thyroiditis, and six had had surgery.

**Table 1:** Data on 66 patients initiating LT4/LT3 combination therapy and participating in a cross-sectional study 5 years later. Data are given as the median (range) or as *n* (%). Bold values indicate statistical significance (*P *<0.05).

	Patients treated with LT4/LT3 combination (*n* = 54)	LT4 treated patients (*n* = 12)*	*P* values
Diagnosis of hypothyroidism			
Year of initiating LT4 treatment	2008 (1985–2017)	2009 (1982–2018)	0.61
Serum TSH^†^	10.5 (3.4–150)^‡^	6.8 (6.6–17)	
Sex (F/M)	52/2	12/0	0.51
Shifting from LT4 to LT4/LT3 combination			
Year of shifting regime	2014 (2012–2018)	2013 (2013–2019)	0.58
TSH at new regime	0.74 (0.01–3.08)	0.84 (0.2–2.2)	0.96
Medication when initiating LT3 treatment			
LT4 dose (μg)	128.6 (71.4–250)	100 (75–200)	0.27
LT3 dose (μg)	7.5(5–20)	5.0(5–10)	**0.050**
LT4/LT3 ratio	16.7 (5–28.6)	17.1(10.0–20.0)	0.43
Cross-sectional study			
Age	56 (40–77)	58 (28–69)	0.81
BMI	29 (20–45)	27 (22–45)	0.69
TSH	0.61 (<0.01–5.28)	0.53 (0.07–3.16)	0.96
Time between T3 initiation and cross-sectional study (years)	5.4 (1.7–10.9)	5.9 (0.71–3.4)	0.80
Medication at cross-sectional study			
T4 dose (μg)	100 (50–200)	100 (50–171.4)	
LT3 dose (μg)	8.75 (2.5–35)		
LT4/LT3 ratio	13.4 (3–37)^§^		
Comorbidity within 10 years before the cross-sectional study			
Depression	7 (13%)	4 (33%)	0.09
Stress	28 (52%)	3 (25%)	0.50
Stress needing sick leave	20 (37%)	3 (25%)	
Known osteoporosis	2 (4%)	0	
Hypertension	5 (9%)	5 (42%)	0.005
Cancer	9 (17%)	1 (8%)	0.12

* LT3 treatment was stopped in these patients due to lack of effect. Back on LT4.

^†^ Data available on 46 patients, 43 of 54 LT4/LT3 treated patients and 3/12 LT4 treated patients.

^‡^ One patient was initiating LT4 on TSH 3.4 but had during follow-up a low dose of LT4 TSH levels above the normal range.

^§^ The difference between T4/T3 ratio when initiating T3 and at the actual study ns (*P* = 0.99).

### Educational level

Eight had less than ten years of education, 25 had shorter education (10–12 years) and 33 had extended education (more than 12 years).

### Thyroid function tests

#### When hypothyroidism was diagnosed

In 46 patients, the exact value of TSH at diagnosis was present; 20 patients (46%) had TSH between upper reference level and 10 mU/L (18 T3 responders) and 26 patients had TSH of more than 10 mU/L at the time of diagnosis (23 T3 responders). Thus, due to only moderately high-serum TSH at diagnosis, most patients probably had subclinical hypothyroidism before initial LT4 was started (22).

When shifting from monotherapy to combined LT4/LT3 treatment, three had TSH below 0.01 mU/L, 12 had TSH between 0.1 and 0.4 mU/L and 51 had TSH within the normal range.

At the time of study, 25 of 66 (38%) had a TSH below the reference range (16 had unmeasurable TSH), one had an elevated TSH, whereas 40 (60%) had a TSH within the normal range. A suppressed TSH was observed in 23 patients (43%) in the T3 responder group and in four patients (33%) in the T3 non-responder group.

#### Medication

When initiating LT4/LT3 combination treatment, the LT3 dose was 7.5 μg (range: 5–20 μg), giving a median LT4/LT3 ratio of 17.

### QoL evaluated by ThyPRO

Overall, QoL and depression scales showed a tendency toward poorer QoL in T3 non-responders compared to T3 responders; however, this difference was not significant when corrected for multiple testing (Table 2).

**Table 2:** QoL measured by ThyPRO for 66 patients initiating LT4/LT3 combination therapy and participating in a cross-sectional study 5 years later.

ThyPRO	Responders (*n* = 54)*	Non-responders (*n* = 12)^†^	*P* values^‡^
Overall QoL impact	25 (0–75)	44 (13–94)	0.036
Tiredness	42 (8–92)	67 (8–92)	0.092
Cognitive complaints	21 (1–85)	29 (7–52)	0.50
Anxiety	10 (1–71)	18 (1–56)	0.58
Depression	14 (0–71)	26 (0–71)	0.063
Emotional susceptibility	21 (7–77)	32 (7–68)	0.15
Impaired social life	0 (0–50)	0 (0–25)	0.86
Impaired daily life	15 (0–62)	19 (0–89)	0.17
ThyPRO composite score	20 (3–73)	27 (3–55)	0.094

* Still on combination therapy.

^†^ Back on LT4.

^‡^ *P* < 0.05 are statistically significant.

**Figure 2:**
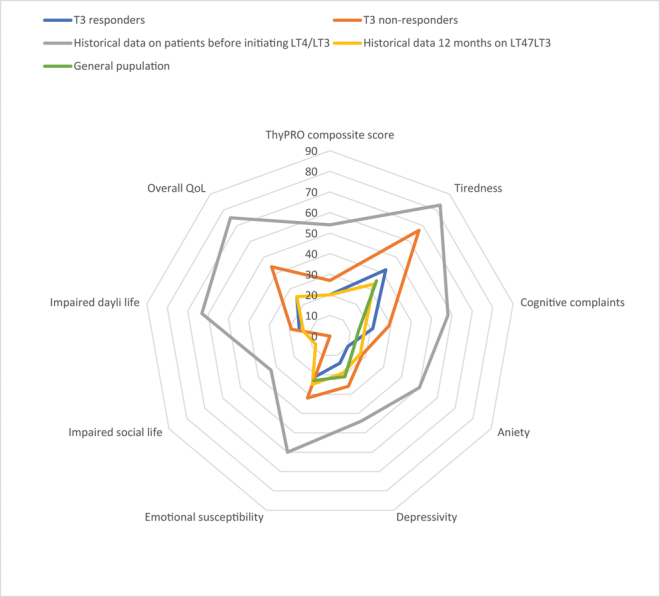
QoL measured by ThyPRO score evaluated 5.4 years after initiating LT4/LT3 combination therapy. T3 responders (*n* = 54, dark blue line) and T3 non-responders (discontinuing LT3 treatment due to lack of effect) (*n* = 12, orange line) compared to historical data before initiating treatment (*n* = 23 gray line) and 12 months after initiating treatment (yellow line) (15)) and the general population (*n* = 739 (18)) (light blue line).

When comparing T3 responders to historical data on a sample of the general population with similar age (18), QoL was at a similar level and much better than compared to historical data on LT4-treated patients with persistent symptoms (15). See Fig. 2.

When comparing QoL in the T3 responders of those with a TSH <0.4 mU/L (38% of the patients) versus TSH ≥0.4 mU/L, a tendency of better scores were seen in anxiety score (10 vs 18, *P* = 0.010), emotional susceptibility score (13 vs 21, *P* = 0.021) and ThyPRO composite score (17 vs 23, *P* = 0.044) in the patients having TSH <0.4; however, not significant when adjusting for multiple testing. (Data shown in Supplement II).

#### Symptoms evaluated by hypothyroid symptoms score

**Figure 3:**
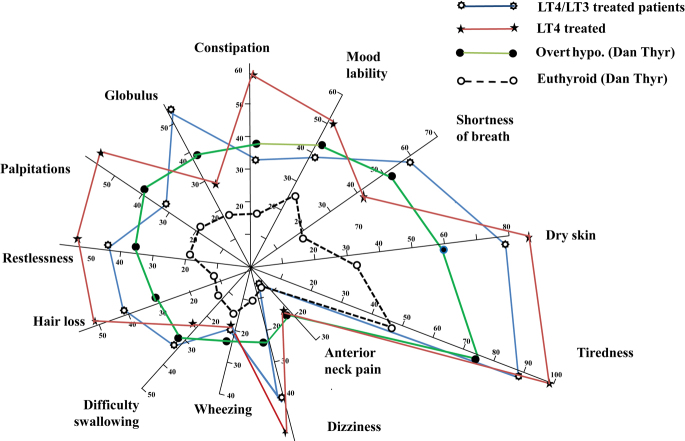
Symptoms score. T3 responders (*n* = 54, blue line) and T3 non-responders (discontinuing LT3 treatment due to lack of effect) (*n* = 12, red line) compared to untreated clinical hypothyroidism (TSH >10 mU/L) (*n* = 108) in a matched patient’s group (green line) and euthyroid controls (*n* = 216) from the Dan Thyr (dotted line) (19, 20, 21).

Both T3 responders and T3 non-responders still had many hypothyroid symptoms. When comparing to data on newly diagnosed hypothyroidism, both T3 responders and T3 non-responders had symptom scores corresponding to symptoms in untreated patients of similar age having overt hypothyroidism (TSH >10) (19) (Table 3 and Fig. 3).

**Table 3:** Symptoms score in 66 patients initiating LT4/LT3 combination therapy and participating in a cross-sectional study 5 years later. Data are presented as percent reporting symptom positivity.

Symptoms	Responders (*n* = 54)*	Non-responders (*n* = 12)^†^	*P* values^‡^
Tiredness	89	100	0.092
Dry skin	78	92	0.27
Shortness of breath	60	40	0.24
Globulus	53	33	0.20
Restlessness	43	58	0.32
Dizziness	43	50	0.64
Hair loss	41	50	0.56
Mood lability	39	50	0.48
Constipation	33	60	0.28
Palpitation	31	58	0.08
Difficulties swallowing	33	25	0.58
Wheezing	20	8	0.33
Anterior neck pain	8	17	0.32

* Still on combination.

^†^ Back on LT4 due to no effect of LT4/LT3.

^‡^ *P* < 0.005 is statically significant.

When comparing symptom scores in the T3 responders, TSH <0.4 mU/L versus TSH ≥0.4, significantly better scores with TSH <0.4 were seen regarding mood lability as 14% answered yes in the group of suppressed TSH compared to 55% in the group with TSH within the normal range (*P* = 0.003), and a tendency was seen regarding restlessness score (*P* = 0.03). (Data shown in Supplement II)

#### Side effects

Two patients had previously been diagnosed with osteoporosis, and one patient demonstrated previously unknown atrial fibrillation at the study visit. None of the T3 responders replied that they had side effects of LT3 treatment related to heart, gastrointestinal, weight, sleep or psychiatric symptoms. In the T3 non-responder group, none of the patients had stopped treatment due to side effects.

#### Overtreatment (defined as TSH <0.4 mU/L)

In this study, 38% (*n* = 25 of 66) of the included patients were overtreated, defined as having a serum TSH <0.4 mU/L. Of these, 24% (*n* = 16) had a serum TSH <0.1 mU/L.

## Discussion

In the current cross-sectional study of hypothyroid patients with persistent symptoms who had previously shifted from LT4 monotherapy to combined LT4/LT3 therapy, 82% stayed on combination treatment after initiation. Many were overtreated (38%). However, treatment was well-tolerated with no side effects, and the responders had high QoL but still experienced many symptoms.

Although there is no strict evidence of better QoL related to LT4/LT3 therapy, it is requested by patients and used by endocrinologists worldwide (2). Online European questionnaires (the Thesis study) included data from 28 countries and 17,247 doctors treating hypothyroid patients. Of these, 40% indicated that they would consider using LT4/LT3 combination treatment in patients with persistent symptoms. There were significant differences between countries, and doctors in countries with high BNP were more likely to use LT4/LT3 therapy than those in countries with low gross national income (GNI) (23).

Requests from patients to have LT4/LT3 combination treatment are often the reason for initiating therapy. A qualitative study from the USA among ATA members describes the discrepancy between guidelines and patient requests, showing that patients’ wishes were reported as a barrier to therapy following guidelines recommending against the use of LT4/LT3 combination treatment (24). In a previous study from our group, we found that the odds of being treated with LT3 or desiccated thyroid extract almost doubled with the highest educational level compared to the lowest (25).

The QoL scores in T3 responders in our study (see Fig. 2) were similar to those in the general population at the same age. An unexpected finding was that when evaluating the hypothyroidism symptom scores, both the T3 non-responders and T3 responders had high scores of hypothyroid symptoms. Previously, it was found that comorbidity by far predicted which subclinical hypothyroid patients had symptoms (21). Without comorbidity, patients with subclinical hypothyroidism had no higher symptom burden compared to euthyroid patients. Patients in the present study, who most probably had subclinical hypothyroidism, reported a high burden of symptoms, and they may be highly self-selected for combination therapy due to other diseases (higher comorbidity). Other diseases may not be alleviated by adding LT3 to LT4 therapy. The symptom scores were similar to those reported in untreated, overt hypothyroid women in the same age group, suggesting that the symptoms in both responders and non-responders are not related to LT3 treatment.

Many studies show that only around 60% of hypothyroid patients (26) have normal TSH during treatment. The dominant problem is usually undertreatment; however, 38% were overtreated in this study. Although the intention was to follow European guidelines (1), the patients tend to be overtreated.

A recent paper has evaluated an online, multinational cross-sectional survey of individuals with self-reported, treated hypothyroidism using the patient health questionnaire-15 (PHQ-15) (*n* = 3516). They assessed the frequency of somatization (associated with distress and high healthcare resource use), previously classified as somatic symptom disorder (SSD). In this population, they found a prevalence of pSSD (defined as a score >10 using the PHQ-15) of 58.6% versus 4–25% in reference populations (27). This could explain the high frequency of symptoms. However, our patient group had a high frequency of comorbidity, especially stress, which could also influence this, making it difficult to compare the studies.

Another explanation for the high rate of hypothyroid symptoms without worse QoL could also be related to increased awareness of these symptoms in this population.

Few studies have evaluated the side effects of LT3 treatment. A large Swedish register-based study compared 11,147 LT3 users (9614 on a combination of LT4 and LT3) median LT3 dose 17 μg (range: 3–26) with 564,314 LT4-only users. No differences were seen in cancer or all-cause mortality; the limitations of that study are relatively young patients in the LT3 user group (median of 46 years old) and a follow-up of only 3 years (12).

A retrospective Korean study evaluated the risk of cardiac failure and stroke in 1434 LT3 users compared to 3908 LT4 users only. They found an increased risk of heart failure (incident rate ratio (IRR) at 1.66) and stroke (IRR: 1.76). The patients using LT3 >52 months had the highest risk. The limitation of this study is the lack of data on T3 dose and s-TSH levels (13).

The major limitation of our present study is that it is a small, unblinded study on a highly selected patient group striving for an effect of the LT4/LT3 combination. However, to our knowledge, no previous studies have described a clinical follow-up in this patient group.

Another limitation is that only 66 of the 147 (45%) patients invited to the study joined the study; most had an effect of T3 (responders), so there may be selection bias toward patients experiencing the impact of the LT4/LT3 treatment. We do not have data on the patients not responding to the invitation as we only had acceptance from the ethical committee to invite the patients by letter; we were not allowed to call the patients or to look up data in the digital journal system for further data.

It could be optimal to look at patients with overt hypothyroidism at diagnosis. However, we wanted to look at actual patients treated in our outpatient clinic, and we believe that these are similar patient populations seen in other outpatient clinics in Europe and the USA. The study’s strength is that it is a real-life study representing a patient group experiencing an effect of treatment as they are met in an outpatient setting.

## Conclusion

Evaluated on QoL, the patients continuing LT4/LT3 treatment experienced a QoL comparable to the background population after four years of treatment, with no side effects. Surprisingly, despite good QoL, they still report as many hypothyroid symptoms as women of the same age with untreated overt hypothyroidism.

## Supplementary materials



## Declaration of interest

The authors declare that there is no conflict of interest that could be perceived as prejudicing the impartiality of the work reported.

## Funding

A grant from the Danish Medicines Agency 2018 supported the work.

## Author contribution statement

BN conceived the study (together with JLC and AC) and wrote the paper. BN and CZJ made the database. CZJ performed data collection. MJ made the statistical analysis. RS analyzed the genetic variations. AC conceived the study. JLC conceived the study. All have taken part in the data management and have read, commented and accepted the final manuscript.
