# Supplementary material for: Long-term outcomes of LT4/LT3 combination treatment for persistent hypothyroid symptoms
Source: Eur Thyroid J. 2025 Mar 4;14(2):e240275. doi: 10.1530/ETJ-24-0275 (PMC11906148; doi:10.1530/ETJ-24-0275)
Supplement: Supplementary file 1 [file supplementary_materials.pdf]

Supplement I.

Data on nucleotide variations (rs17606253 in the Solute Carrier Family 16 Member 10 (*SLC16A10)* gene and three SNPs ((rs225014 (Thr92Ala), rs12885300 (ORFa-Gly3Asp) and rs225015)) in the type 2 deiodinase (*DIO2) g*ene in 66 patients initiating LT4/LT3 combination treatment due to persistent hypothyroid symptoms on LT4 treatment.

Methods:

Genetics: DNA was extracted from EDTA-stabilized whole blood using the Maxwell 16 Blood DNA Kit on the Maxwell RSC Instrument (Promega, US). SNP (single-nucleotide polymorphism) genotyping was performed on 66 patients, and 88 randomly selected healthy adult Danish blood donors were recruited from the Blood Bank at Aalborg University Hospital, Aalborg, Denmark.
Using predesigned TaqMan SNP Genotyping Assays (Applied Biosystems, Foster City, CA, USA), four single nucleotide variations were genotyped: rs17606253 in the Solute Carrier Family 16 Member 10 (*SLC16A10)* gene and three SNPs ((rs225014 (Thr92Ala), rs12885300 (ORFa-Gly3Asp) and rs225015)) in the type 2 deiodinase (*DIO2) g*ene [1,2].

Results: When comparing the selected gene variations (rs225014 (Thr92Ala), rs12885300 (ORFa-Gly3Asp), rs225015 in the *DIO2 g*ene, or rs1760625 in the *SLC16A10* gene), no significant difference was seen related to ThyPRO score or symptoms score regarding responders versus non-responders or subclinical versus overt hypothyroidism at the time of diagnosis. The low rate of non-responders makes it difficult to draw conclusions from these data.

Data on rs225014 (Thr92Ala) are shown in Table 1 and 2.

The genetic variations in the study population were not higher than those of Danish blood donors (data not shown).

Discussion: The gene variation D2 Thr92 Ala has been described as responsible for a decreased deiodination of T4 to T3 on a cellular level and suggested to explain a decreased Qol and persistent symptoms[3]. A post-study analysis of our randomized controlled trial on LT4/LT3 vs LT4 showed that patients with genetic variations related to the deiodinase 2 gene (rs225014 (Thr92Ala), rs12885300 (ORFa-Gly3Asp), rs225015) or linked to the membrane transporter *SLC16A10* (rs17606253*)* had a preference for T4/T3 combination treatment. A recent large-scale study from the UK Biobank, which evaluated DIO2 and *SLC16A10* gene variations (rs225014, rs225015, rs12885300, and rs17606253) in 18,761 LT4-treated patients and 360,534 controls could not explain changed psychological well-being, cognitive function, or cardiovascular risk factors in LT4-treated patients or controls [4], which is in accordance with other smaller population-based studies [5,6] . In the current study we included an insufficient number of patients to evaluate the impact of these genetic variations.

References:

1. Steffensen R, Baech J, Nielsen KR. Allelic Discrimination by TaqMan-PCR for Genotyping of Human Neutrophil Antigens. Methods Mol Biol Clifton NJ. 2015;1310:205–12.
2. Carlé A, Faber J, Steffensen R, Laurberg P, Nygaard B. Hypothyroid Patients Encoding Combined MCT10 and DIO2 Gene Polymorphisms May Prefer L-T3 + L-T4 Combination Treatment - Data Using a Blind, Randomized, Clinical Study. Eur Thyroid J. 2017 Jul;6(3):143–51.
3. Panicker V, Cluett C, Shields B, Murray A, Parnell KS, Perry JRB, et al. A common variation in deiodinase 1 gene DIO1 is associated with the relative levels of free thyroxine and triiodothyronine. J Clin Endocrinol Metab. 2008 Aug;93(8):3075–81. .
4. Jensen CZ, Isaksen JL, Ahlberg G, Olesen MS, Nygaard B, Ellervik C, et al. Association of DIO2 and MCT10 Polymorphisms with Persistent Symptoms in LT4-Treated Patients in UK Biobank. J Clin Endocrinol Metab. 2023 Sep 22;dgad556.
5. Appelhof BC, Peeters RP, Wiersinga WM, Visser TJ, Wekking EM, Huyser J, et al. Polymorphisms in type 2 deiodinase are not associated with well-being, neurocognitive functioning, and preference for combined thyroxine/3,5,3'-triiodothyronine therapy. J Clin Endocrinol Metab. 2005 Nov;90(11):6296–9.
6. Wouters HJCM, van Loon HCM, van der Klauw MM, Elderson MF, Slagter SN, Kobold AM, et al. No Effect of the Thr92Ala Polymorphism of Deiodinase-2 on Thyroid Hormone Parameters, Health-Related Quality of Life, and Cognitive Functioning in a Large Population-Based Cohort Study. Thyroid. 2017 Feb;27(2):147–55

Table 1: Results genetic variation rs 225014 (Thr92Ala) related to ThyPRO

Stratified only on effect = yes

	**median**	n	p
Hypothyroid symptoms			
T/T (wildtype)	31,00	20	
C/T	25,00	29	
C/C	38,00	5	
Total	25,00	54	0.666
Tiredness			
T/T (wildtype)	42,00	20	
C/T	42,00	29	
C/C	67,00	5	
Total	42,00	54	0.512
Cognitive complaints			
T/T (wildtype)	25,00	20	
C/T	21,00	29	
C/C	14,00	5	
Total	21,00	54	0.240
Anxiety			
T/T (wildtype)	18,0	20	
C/T	10,0	29	
C/C	1,0	5	
Total	10,0	54	0.236
Depresssivity			
T/T (wildtype)	14,0	20	
C/T	14,0	29	
C/C	14,0	5	
Total	14,0	54	0.813
Emotional susceptibility			
T/T (wildtype)	24,5	20	
C/T	13,0	29	
C/C	21,0	5	
Total	21,0	54	0.889
Impaired social life			
T/T (wildtype)	0,0	20	
C/T	0,0	29	
C/C	0,0	5	
Total	0,0	54	0.710

Impaired daily life			
T/T (wildtype)	15,0	20	
C/T	15,0	29	
C/C	0,0	5	
Total	15,0	54	0.405
Thypro composite score			
T/T (wildtype)	26,0	20	
C/T	18,0	29	
C/C	17,0	5	
Total	19,5	54	0.766

Stratified only on effect = no

	median	n	
Hypothyroid symptoms			
T/T (wildtype)	50,00	5	
C/T	31,50	6	
C/C	69,00	1	
Total	44,00	12	0.587
Tiredness			
T/T (wildtype)	67,00	5	
C/T	46,00	6	
C/C	92,00	1	
Total	67,00	12	0.196
Cognitive complaints			
T/T (wildtype)	21,00	5	
C/T	25,50	6	
C/C	37,00	1	
Total	29,00	12	0.820
Angst			
T/T (wildtype)	18,0	5	
C/T	14,0	6	
C/C	26,0	1	
Total	18,0	12	0.674
Depresssivity			
T/T (wildtype)	37,0	5	
C/T	18,0	6	
C/C	54,0	1	
Total	25,5	12	0.308
Emotional susceptibility			
T/T (wildtype)	36,0	5	
C/T	24,5	6	

C/C	44,0	1	
Total	32,0	12	0.873
Impaired social life			
T/T (wildtype)	0,0	5	
C/T	0,0	6	
C/C	25,0	1	
Total	0,0	12	0.175
Impaired daily life			
T/T (wildtype)	22,0	5	
C/T	0,0	6	
C/C	62,0	1	
Total	18,5	12	0.382
Thypro composite score			
T/T (wildtype)	25,0	5	
C/T	25,0	6	
C/C	51,0	1	
Total	26,5	12	0.242

Table 2: **Results genetic variation rs 225014 (Thr92Ala) related to Symptom score.**

	rs225014			
Globulus	Yes	no	N	p
T/T (wildtype)	12	13	25	
C/T	19	16	35	
C/C	2	4	6	
Total	33	33	66	0.618
Difficulties swallowing				
T/T (wildtype)	9	16	25	
C/T	10	25	35	
C/C	2	4	6	
Total	21	45	66	0.828
Anterior neck pain				
T/T (wildtype)	3	22	25	
C/T	3	31	34	
C/C	0	6	6	
Total	6	59	65	0.655
Wheezing				
T/T (wildtype)	2	23	25	

C/T	7	28	35	
C/C	3	3	6	
Total	12	54	66	0.052
Shortness of breath				
T/T (wildtype)	13	12	25	
C/T	20	14	34	
C/C	4	2	6	
Total	37	28	65	0.767
Restlessness				
T/T (wildtype)	11	14	25	
C/T	17	18	35	
C/C	2	4	6	
Total	30	36	66	0.773
Palpitations				
T/T (wildtype)	7	18	25	
C/T	13	22	35	
C/C	4	2	6	
Total	24	42	66	0.207
Mood lability				
T/T (wildtype)	11	14	25	
C/T	13	22	35	
C/C	3	3	6	
Total	27	39	66	0.775
Constipation				
T/T (wildtype)	9	16	25	
C/T	11	24	35	
C/C	4	2	6	
Total	24	42	66	0.253
Tiredness				
T/T (wildtype)	23	2	25	
C/T	31	4	35	
C/C	6	0	6	
Total	60	6	66	0.648
Hair loss				
T/T (wildtype)	11	14	25	
C/T	15	20	35	
C/C	2	4	6	
Total	28	38	66	0.891
Dry skin				

T/T (wildtype)	22	3	25	
C/T	26	9	35	
C/C	5	1	6	
Total	53	13	66	0.412
Vertigo				
T/T (wildtype)	12	13	25	
C/T	13	22	35	
C/C	4	2	6	
Total	29	37	66	0.353

Supplement II

Data on patients initiating LT4/LT3 combination therapy and participating in a Cross-sectional study 4 years later (n=52).

Comparing patients still on LT4/LT3 combination therapy having suppressed TSH <0.4mU/l compared to patients having TSH≥ 0.4 mU/l.

Table 1a: Data on patients initiating LT4/LT3 combination therapy and participating in a Cross-sectional study 5 years later (n=52).

Comparing patients still on LT4/LT3 combination therapy having suppressed TSH (<0.4mU/l) compared to patients having TSH≥ 0.4 mU/l .

Median and range

	**LT4/LT3 treated patients still on a combination. TSH<0.4 at cross-sectional study (n= 21)**	**LT4/LT3 treated patients still on a combination TSH≥ 0.4 at cross-sectional study (n= 33)**	p – values
**Diagnose of hypothyroidism**			
Year of initiating LT4 treatment	2009 (1993-2017)	2008 (1985-2017)	0.37
TSH at diagnosis	15 (n=19) (3.4-102)	7.4 (n= 19) (4.0-150)	0.28
Sex (F/M)	21/0	31/2	0.25
**Shifting from LT4 to LT4/LT3 combination**			
Year of shifting regime	2014 (2012-2018)	2014 (2012-2018)	0.21

TSH at new regime (median, range)	0.77 (0.02-3.08)	0.71 (0.01-2.90)	0.76
**Medication, when initiating L-T3 treatment**			
L-T4 dose (µg)	120 (75-250)	143 (71-200)	0.89
L-T3 dose (µg)	7.5 (5-10)	7.5 (5-20)	0.87
L-T4/L-T3 ratio	17.1 (5-23)	16.7 (5-29)	0.34
**Cross-sectional study**			
Age (median, range)	56 (41-68)	54 (40-77)	0.61
BMI (median, range)	27 (20-41)	30 (22-45)	0.11
TSH (median, range)	0.04 (<0.01-0.36)	0.85 (0.41-5.28)	-
The time between initiation of T3 to the cross-sectional study (years)	5.4 (3.0-6.5)	5.3 (1.7-10.9)	0.83
**Medication, at cross-sectional study**			

T4 dose at cross-sectional study (µg)	110<br>(71-200)	100<br>(50- 186)	0.38
T3 dose at cross-sectional study (µg)	10<br>(5-35)	7.5<br>(2.5-10)	0.02
L-T4/L-T3 ratio	12.1<br>(3-37)	14.3<br>(7-37)	0.10

Table 2b :QoL measured by ThyPRO 54 patients still on L4/LT3 combination therapy comparing patients having suppressed TSH 8>0.4 mu/l) to patients having TSH ≥ 0.4mU/l.

	**TSH<0.4mU/l (n=21)**	**TSH ≥0.4 mU/l (n=33**	**Sign: if < 0.005**
**ThyPRO**	**Median (range)**	**Median (range)**	
Overall QoL impact of hypothyroidism	25 (0-75)	31(0-69)	0.55
Tiredness	37(17-67)	50(8-92)	0.09
Cognitive complaints	21(1-60)	21(1-85)	0.21
Anxiety	10(1-26)	18(1-71)	0.01
Depression	14(0-45)	22(0-71)	0.50
Emotional susceptibility	13(7-17)	21(7-77)	0.02
Impaired social life	0(0-62)	0(0-50)	0.27
Impaired daily life	0(0-62)	15(0-62)	0.30
ThyPRO composite score	17 (-3-35)	23(6-67)	0.04

Table 3b: **Symptom score in 54 patients still on L4/LT3 combination therapy comparing patients having suppressed TSH 8>0.4 mu/l) to patients having TSH > 0.4mU/l.**

	Responders TSH<0.4mU (n=21)	Responders TSH≥0.4mU/l (n= 33)	Sign: if < 0.005
**Symptoms**	**% yes**	**% yes**	
Tiredness	90	88	0.08

Dry skin	76	79	0.82
Shortness of breath	48	69	0.12
Globulus	57	52	0.69
Restlessness	24	55	0.03
Dizziness	48	39	0.56
Hair loss	48	36	0.41
Mood lability	14	55	0.003
Constipation	43	27	0.26
Palpitation	24	36	0.33
Difficulties swallowing	38	30	0.55
Wheezing	19	21	0.66
Anterior neck pain	10	6	0.24
